# Three new species of *Cosmocerca* Diesing, 1861 (Nematoda: Cosmocercidae) parasitising frogs *Cacosternum boettgeri* Boulenger, 1882, *Kassina senegalensis* Dumeril and Bibron, 1841 and *Phrynomantis bifasciatus* Smith, 1847 from South Africa

**DOI:** 10.1007/s00436-021-07390-7

**Published:** 2022-01-19

**Authors:** F. Harnoster, L. H. du Preez, R. Svitin

**Affiliations:** 1 African Amphibian Conservation Research Group, Unit for Environmental Sciences and Management, North-West University, Potchefstroom, South Africa; 2 South African Institute for Aquatic Biodiversity, Private Bag 1015, Grahamstown, 6140 South Africa; 3 Department of Invertebrate Fauna and Systematics, I. I. Schmalhausen Institute of Zoology, 15 Bogdan Khmelnytskyi Street, Kyiv, 01030 Ukraine

**Keywords:** Africa, Anura, Nematoda, Cosmocercidae, *Cosmocerca*, *Cacosternum boetgerri*, *Kassina senegalensis*, *Phrynomantis bifasciatus*

## Abstract

Cosmocercid nematodes have been documented with much criticism due to the numerous inaccurate descriptions, redescriptions and synonymisation of found species. This is due to indistinguishable characters of females and the lack of male specimens found. Consequently, the species *C. ornata* is the most commonly found species worldwide and the only species of the genus reported in South Africa. In the present study, we found *Cosmocerca* in three different amphibian species, namely *Cacosternum boettgeri*, *Kassina senegalensis* and *Phrynomantis bifasciatus*. Based on differences in the shape of the gubernaculum and number of papillae, the found nematodes were assigned to three new species namely *C.* *daly* n. sp., *C. monicae* n. sp. and *C. makhadoensis* n. sp. Descriptions of species are followed by pairwise and phylogenetic analysis of partial ITS-28S sequences. All three species were found only in their host types from distant localities. Therefore, we hypothesise that host specificity of *Cosmocerca* from South African amphibians might be rather high and that the presence of *C. ornata* throughout South Africa is rather doubtful.

## Introduction

Nematodes of the family Cosmocercidae are one of the most abundant groups of nematodes that parasitise the digestive tract of cold-blooded vertebrates worldwide. They have a high biodiversity and are distinguished generally by the morphology of the male genital system (Baker 1987; Martinez and Maggenti 1989; Bursey et al. 2011). There are three subfamilies: Austraplectaninae, parasitising amphibians in Australia; Maxvachoniae, parasitising amphibians in Australo-Papuan Region (Bursey et al. 2011); and Cosmocercinae, reported from amphibians and rarely reptilians worldwide. Of these, only Cosmocercinae are known from the African continent. Of the subfamily Cosmocercinae, the most abundant genera are *Aplectana* Railliet and Henry, 1916 and *Cosmocerca* Diesing, 1861(Baker 1987). The only specific study on cosmocercids in southern Africa was done by Baker (1981), based on material collected from several amphibian species in South Africa, Zimbabwe and Namibia. In that material, the author identified *Aplectana chamaeleonis* Baylis, 1929 from *Sclerophrys capensis* (Tschudi, 1838) (reported as *Bufo rangeri*), *Vandijkophrynus angusticeps* (Smith, 1848) (reported as *Bufo angusticeps*), *Amietia delalandii* (Duméril and Bibron, 1841) (reported as *Rana angolensis*), *Ptychadena oxyrhynchus* (Smith, 1849) and *Cacosternum capense* Hewitt, 1925; *A. macintoshii* Stewart, 1914 from *Schismaderma carens* (Smith, 1848) (reported as *Bufo carens*), *Sclerophrys gutturalis* (Power, 1927) (reported as *Bufo gutteralis*), *Sclerophrys pusilla* (Mertens, 1937) (reported as *Bufo maculatus*), *Sclerophrys garmani* (Meek, 1897) (reported as *Bufo garmani*), *Phrynomantis bifasciatus* (Smith, 1847) (reported as *Phrynomerus bifasciatus*) and *Breviceps adspersus* Peters, 1882. Baker (1981) also described two new species of *Aplectana*, namely *A. degraaffi* Baker 1981 from *Breviceps sylvestris* FitzSimons, 1930 and *A. capensis* Baker 1981 from *Breviceps rosei* Power, 1926. Of the genus *Cosmocerca*, Baker (1981) identified all specimens found from *Capensibufo rosei* (Hewitt, 1926) (reported as *Bufo rosei*), *S. capensis*, *S. gutturalis*, *S. pusilla*, *S. garmani*, *Mertensophryne anotis* (Boulenger, 1907) (reported as *Bufo anotis*), *Ptychadena anchietae* (Bocage, 1868), *Ptychadena porosissima* (Steindachner, 1867), *Kassina senegalensis* Dumeril and Bibron, 1841 and *Cacosternum namaquense* Werner, 1910 as *C. ornata* Dujardin, 1845. *Cosmocerca ornata* was initially described from the frogs *Rana temporaria* L. and *Pelophylax esculentus* L. (reported as *Rana esculenta*) in Rennes, France (Dujardin, 1845). Subsequently, the species was identified from various amphibian and reptilian hosts throughout Europe, Asia and Africa (Bursey et al. 2011). The latest redescription of *C. ornata* was provided in 2019 (Sou et al. 2019); however, the authors only used the material collected from the dicroglossid frogs *Hoplobatrachus crassus* (Jerdon, 1853) and *Euphlyctis cyanophlyctis* (Schneider, 1799) in India and without comparison with the material type.

Numerous inaccurate descriptions, redescriptions and synonymisation of found species, accompanied by the lack of molecular data, resulted in *C. ornata* being the most commonly found species parasitising amphibians and reptiles in Europe, Asia and Africa (Baker 1981; Halajian et al. 2013; Bursey et al. 2011; Sou et al. 2014). Therefore, the diversity of *Cosmocerca* in South Africa is still limited to one species (Skrjabin et al. 1961; Baker 1981; Sou et al. 2019).

In the present study, nematodes of the genus *Cosmocerca* were collected in Potchefstroom (North-West Province) and Makhado (Limpopo Province), South Africa, from the frogs *Cacosternum* *boettgeri* Boulenger, 1882, *Kassina senegalensis* and *Phrynomantis bifasciatus*. Found specimens appeared to be clearly different from *C. ornata*, each other and from other species of the genus. They were thus assigned to the new species *C.* *daly* n. sp., *C.* *monicae* n. sp. and *C. makhadoensis* n. sp. Descriptions of all three species, followed by line drawings, photomicrographs and molecular data of ITS-28S region are presented herein.

## Materials and methods

In total, 97 frogs (59 *C. boetgeri*, 19 K*. senegalensis* and 19 *P. bifasciatus*) were used in the present study. Host specimens were collected in January, March and December 2019. Amphibians were placed in separate plastic bags containing water and some damp vegetation. At the field station, the water was examined and host specimens that were releasing larvae in the water were processed. Amphibians were anaesthetised with tricaine ethyl-4-aminobenzoate (MS222) and subsequently euthanised through cutting the spine and destroying the brain according to the standard operating procedure (NWU-00492–16-A5) and dissected. Collected nematodes were fixed in hot 70% ethanol and subsequently stored in 70% ethanol for future examinations.

For morphological studies, nematode specimens were placed in distilled water for about 15 min followed by 5 min in lactophenol, then studied under the light microscope on temporary mounts. Apical sections, as well as tail fragments, were prepared manually with a thin razor. Photomicrographs and measurements were taken using ZEISS Z2 and Nikon E800 compound microscopes. For scanning electron microscopy (SEM), the nematodes were dehydrated in a graded ethanol series, dried using hexamethyldisilazane, mounted on stubs, coated with gold and examined using a Phenom Pro SEM microscope.

For molecular analyses, middle segments of the males (one of each species) were used. DNA was extracted using the ZYMO ZR tissue and insect DNA miniprep extraction kit following the protocol recommended by the manufacturer. The ITS-28S region was amplified using the primer pair rift (5’-GCG GCT TAA TTT GAC TCA ACA CGG-3’) and 1500R (5’-GCT ATC CTG AGG GAA ACT TCG-3’) and the thermocycling profile as follows: 2 min denaturation at 94 °C, 40 cycles of 95 °C for 45 s, 54 °C for 45 s, 75 °C for 3 min and 1 cycle at 72 °C for 7 min for extension (Tkach et al. 2014). Unpurified PCR products were sent to a commercial sequencing company, Inqaba Biotechnical Industries (Pty) Ltd (Pretoria, South Africa), where sequences were obtained using BigDye® Terminator v3.1 Cycle Sequencing on an ABI3500XL sequencer. DNA products were sequenced in both directions using pairs of PCR primers and additional internal primers: ITS4 (5’-TCC TCC GCT TAT TGA TAT GC-3’), 300R (5’-CAA CTT TCC CTC ACG GTA CTT G-3’), ITS5 (5’- GGA AGT AAA AGT CGT AAC AAG G-3’) and ECD2 (5’-CTT GGT CCG TGT TTC AAG ACG GG-3’). Contiguous sequences were assembled and edited using Geneious Prime software (https://www.geneious.com). The pairwise analyses (*p*-distance and the number of difference) were performed using Mega (V. 7.0) software. For the phylogenetic analysis, three newly obtained sequences plus seven retrieved from GenBank were aligned using the ClustalW tool in the MEGA v. 9.0 software and trimmed. The final alignment of the ITS-28S region comprised 739 bp. *Cruzia americana* Maplestone, 1930 was selected as the outgroup using the basic alignment searching tool (BLAST). The GTR + G + I nucleotide substitution model was estimated as the best-fitting model prior to analyses using jModelTest (V. 2.1.2). Bayesian inference analysis was run using MrBayes v. 3.2.2 software.

In total, 87 nematode specimens were collected, 35 (five males and 30 females), 18 (four males and 14 females) and 34 (four males and 30 females). Measurements in the text are given as ranges followed by mean values in parentheses and holotype or allotype measurements in square brackets. All measurements are presented in micrometres unless indicated otherwise.

## Results

### Species description

#### *Cosmocerca daly* n. sp.

*General*. Body small, stout, attenuated anteriorly. Mouth triangular with three lips, dorsal lip bearing two prominent cephalic papillae, two ventro-lateral lips bearing each one cephalic papilla and an amphid, with eight body papillae surrounding the cephalus. Three lips opening into oesophagus. Oesophagus divided into three parts: pharynx, cylindrical corpus and oesophageal bulb. Nerve ring encircling oesophagus at level of its mid-length. Excretory pore anterior to beginning of oesophageal bulb. Lateral alae beginning anterior to level of nerve ring and terminating anterior to cloaca. Tail rounded evenly narrowing with short process on tip; straight in females and curved ventrally in males.

**Fig. 1:**
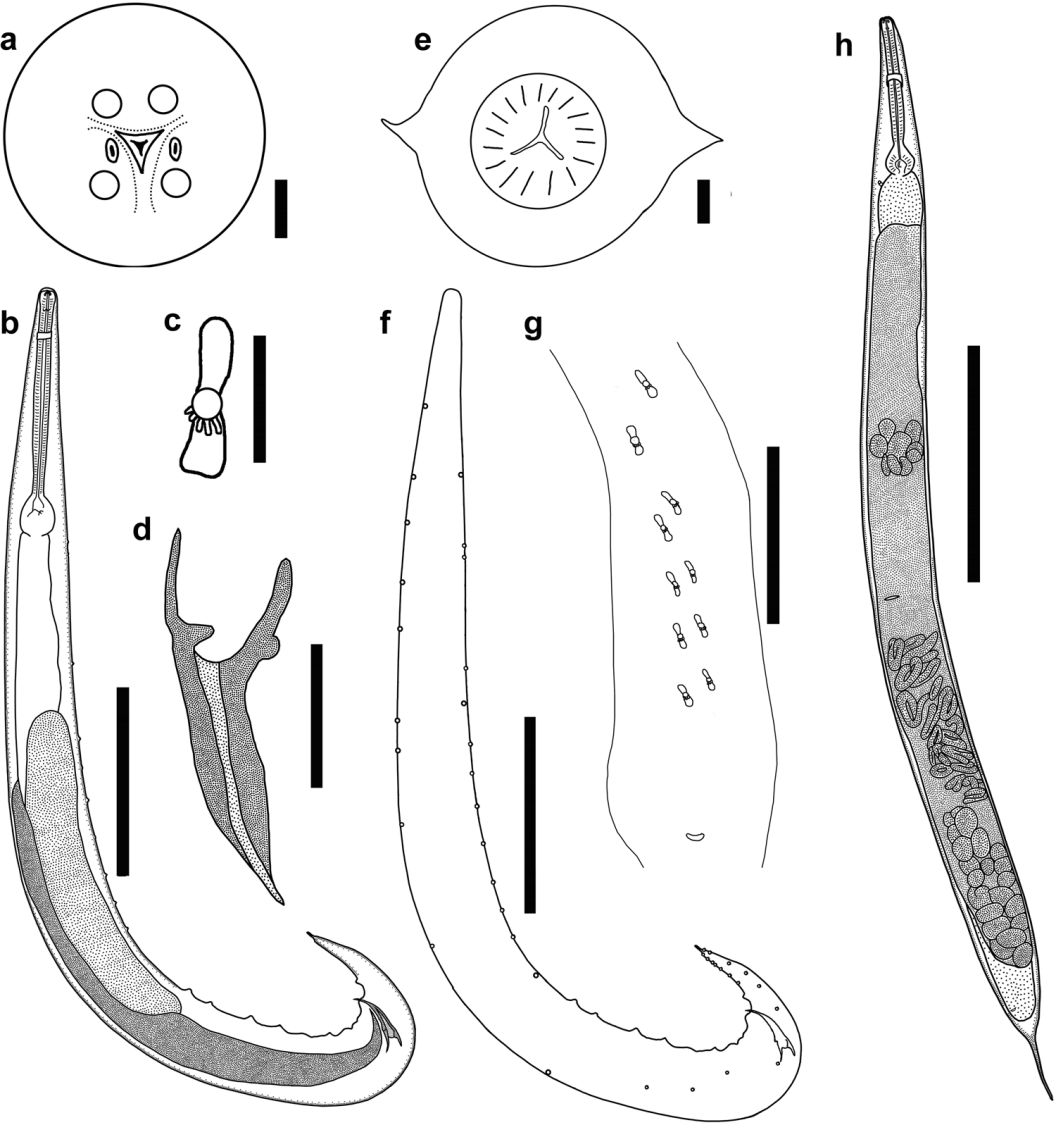
*Cosmocerca daly* n. sp. from *Cacosternum boettgeri*, line drawings. **a** Apical section, female. **b** Full body, male, lateral view. **c** Plectane, ventral view. **d** Gubernaculum, ventral view. **e** transverse section at oesophageal bulb level, female. **f** Arrangements of somatic papillae, male, lateral view. **g** Posterior end of male, ventral view. **h** Full body, female, lateral view. Scale bars: a, c, e – 20 µm; b, d, f, g – 100 µm; h – 1000 µm

*Male*. Measurements based on holotype and four paratypes. Body (Fig. 1b) 1.0–2.7 (1.6) [1.4] mm long, 72–198 (93) [95] wide at mid-body level. Lateral alae beginning at 29–37 (33.5) [30] from apex. Oesophageal pharynx 11–31 (19) [22] long and 13–21 (15) [14] wide; corpus 198–379 (251) [198] long and 20–36 (24) [20] wide; oesophageal bulb 43–81 (56) [47] long and 42–77 (51) [42] wide. Nerve ring at 104–159 (111) [105] and excretory pore at 220–387 (288) [245] from apex. Tail 121–146 (132) [146] long, bearing short process at tip 11–22 (18) [18] long. Gubernaculum (Fig. 1d) 60–89 (73) [89] long, V-shaped, with well sclerotised edges, bearing hook-like structures on its margins. Spicules equal in shape, evenly narrowing with sharpened tips, poorly sclerotised, observed only while dissected. Left spicule 61–98 (77) [84] long, right one 61–102 (80) [86] long. Fourteen pairs of post-cloacal papillae (Fig. 1f) and five pairs of plectanes (Fig. 1c, g) observed in tail region.

*Female*. Measurements based on allotype and 29 paratypes. Body (Fig. 1h) 2–6 (3.7) [4.7] mm long, 74–323 (225) [323] wide at mid-body level. Lateral alae (Fig. 1e) 42–188 (84) [127] from apex. Oesophageal pharynx 17–42 (27) [42] long and 20–41 (31) [38] wide; corpus 240–433 (296) [433] long and 27–47 (34) [47] wide; oesophageal bulb 70–120 (82) [120] long and 62–128 (78) [128] wide. Nerve ring at 117–214 (171) [212] and excretory pore at 206–539 (371) [487] from apex. Vulva at 0.6–2.9 (1.8) [2.5] mm from anterior end of body, small, transversely slit, lips poorly developed, located around the mid-body level (about 46% of body length). Tail 138–408 (289) [408] long, slightly rounded bluntly with an elongated end.

### Taxonomic summary

Family Cosmocercidae Travassos, 1925.

Subfamily Cosmocercinae Railliet, 1916.

Genus *Cosmocerca* Diesing, 1861.

Species: *C. daly* n. sp.

Type-host: Boettger’s dainty frog *Cacosternum boettgeri* (Amphibia: Anura: Microhylidae).

Type-locality: Daly farm, Potchefstroom, North-West Province, South Africa: Coordinates: − 26.650324 / 27.062422.

Type-material: Holotype (male, [665]), allotype (female, [666]), paratypes (four males, 30 females, [667–695]) deposited in the National Museum Parasite Collection (Bloemfontein, South Africa).

Site in host: Large intestine.

Infection parameters: intensity: 1– 24 (7.9); prevalence: 27% (16 of 59 were infected); abundance: 2.1

Etymology: In recognition for support by the Daly family.

*Remarks.*

The new species belongs to the genus *Cosmocerca* due to the possession of plectanes, numerous papillae along the body and sexual dimorphism, since males are half the size of females (Baker 1980).

Hitherto, 30 species of the genus *Cosmocerca* are considered valid of which *C.* *ornata* is the only species recorded in Southern Africa. *Cosmocerca* *daly* n. sp. evidently differed from *C. ornata* in the shape of the gubernaculum, possessing prominent hook-like structures on its margins, in contrast to the simple Y-shaped gubernaculum in *C. ornata*. Moreover, mature females of *C.* *daly* n. sp. have an excretory pore located posterior to the level of oesophageal bulb, whereas it is depicted anterior to the level of oesophageal bulb in *C. ornata*.

**Fig. 2:**
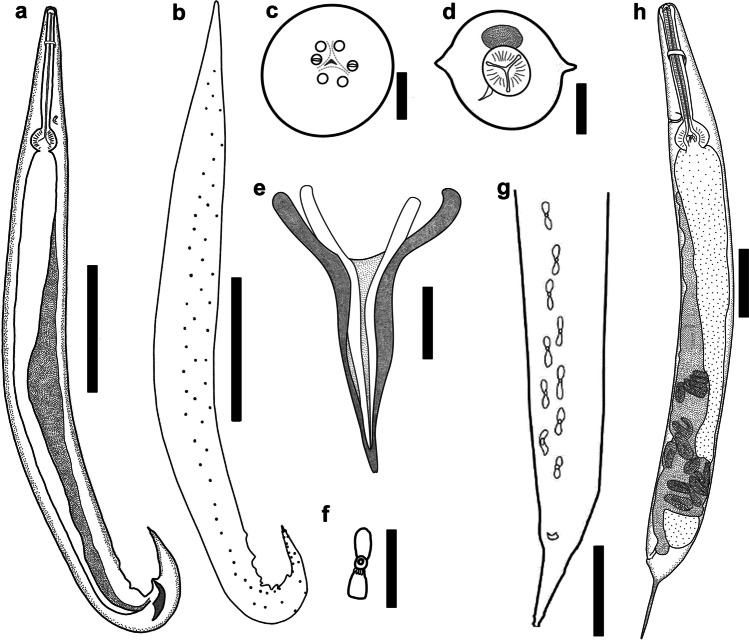
*Cosmocerca monicae* n. sp. from *Kassina senegalensis*, line drawings. **a** Full body, male, lateral view. **b** Arrangements of somatic papillae, male, lateral view. **c** Apical section, female. **d** Transverse section at level of oesophageal bulb, female. **e** Gubernaculum, ventral view. **f** Plectane, ventral view. **g** Posterior end of male, ventral view. **h** Full body, female, lateral view. Scale bars: a, b, g, h – 100 µm; c–f – 20 µm

**Fig. 3:**
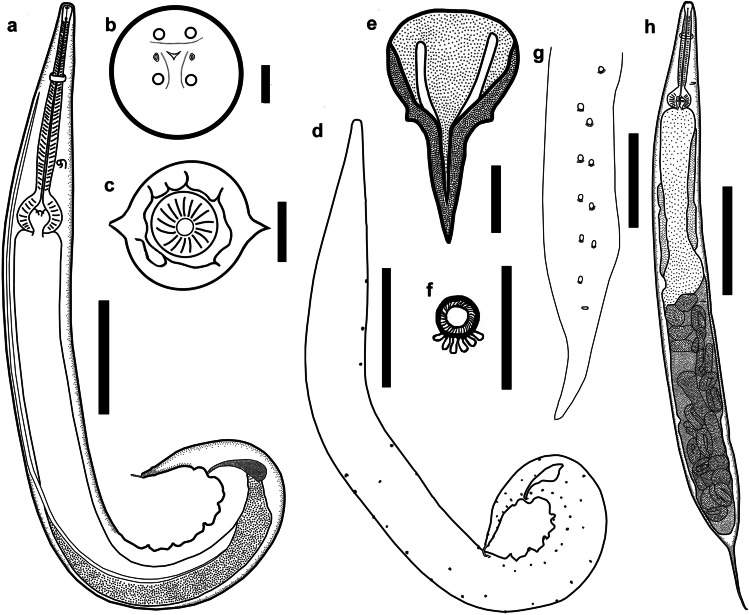
*Cosmocerca makhadoensis* n. sp. from *Phrynomantis bifasciatus*, line drawings. **a** Full body, male, lateral view. **b** Apical section, female. **c** Transverse section at level of oesophageal bulb, female. **d** Arrangements of somatic papillae male, lateral view. **e** Gubernaculum, ventral view. **f** Plectane, ventral view. **g** Posterior end of male, ventral view. **h** Full body, female, lateral view. Scale bars: a, d, g, h – 100 µm; b–e, f – 20 µm

**Fig. 4:**
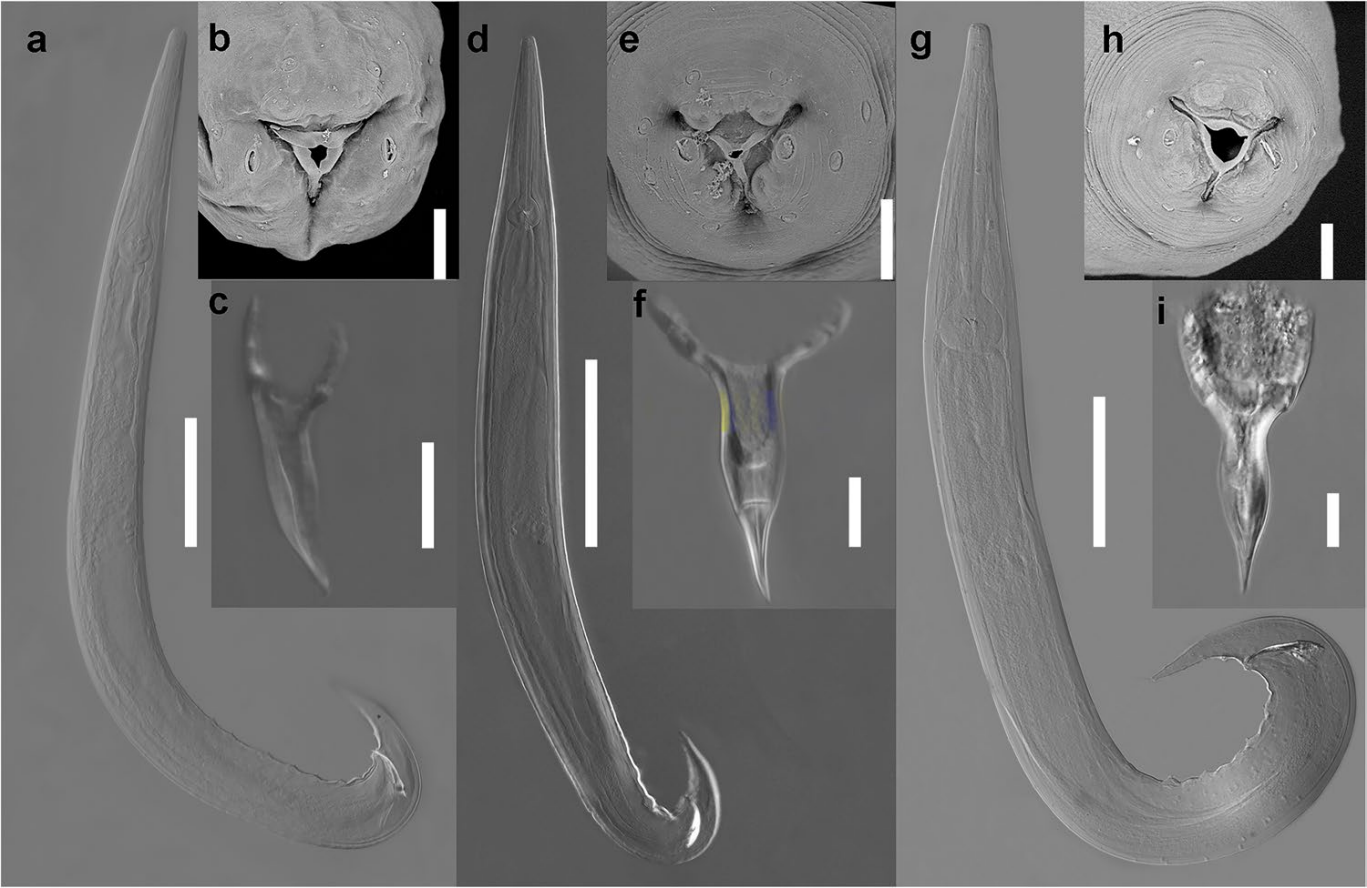
Photomicrographs of three species of *Cosmocerca*. **a–c** *Cosmocerca daly* n. sp. **a** Full body, male, lateral view. **b** *En face* view, female, SEM image. **c** Gubernaculum, ventral view. **d–f** *Cosmocerca* *monicae* n. sp. **d** Full body, male, lateral view. **e** *En face* view, female, SEM image. **f** Gubernaculum, ventral view. **g–i** *Cosmocerca* *makhadoensis* n. sp. **g** Full body, male, lateral view. **h** *En face* view, female, SEM image. **i** Gubernaculum, lateral view. Scale bars: a, d, g – 100 µm; b, c, e, f, h, i – 20 µm

*Cosmocerca daly* n. sp. differs from *C. monicae* n. sp. in the shape of the gubernaculum: *C.* *daly* n. sp. (Figs. 2, 3 and 4a–c) has a V-shaped gubernaculum with hook-like structures on the margins, while *C.* *monicae*. n. sp. (Fig. 4d–f) has a Y-shaped gubernaculum without hook-like structures. *Cosmocerca* *daly* n. sp. also differed from *C. monicae* n. sp. by possessing14 pairs of post-cloacal papillae, whereas *C. monicae* n. sp. has 13 pairs. Additionally, mature females of *C.* *daly* n. sp. have an excretory pore posterior to level of oesophageal bulb, whereas it is depicted anterior to the level of oesophageal bulb in *C. monicae* n. sp.

*Cosmocerca daly* n. sp. also differs from *C. makhadoensis* n. sp. in the shape of the gubernaculum: *C.* *daly* n. sp. (Fig. 4a–c) has a V-shaped gubernaculum with hook-like structures on the margins, whereas *C. makhadoensis* n. sp. (Fig. 4g–i) has a club-shaped gubernaculum without hook-like structures. Additionally, *C.* *daly* n. sp. possesses somatic papillae located only on the lateral sides of the body, while the somatic papillae in *C. makhadoensis* n. sp. were found evenly spread over the entire body, including dorsal and ventral sides. Moreover, the plectanes of *C.* *daly* n. sp. are clearly visible on the lateral and ventral views, whilst the plectanes of *C. makhadoensis* n. sp. have almost transparent edges and are almost inconspicuous in the ventral view. Mature females of *C.* *daly* n. sp. have an excretory pore located posterior to the level of oesophageal bulb, whereas it is depicted anterior to the level of the oesophageal bulb in *C. makhadoensis* n. sp.

#### *Cosmocerca monicae* n. sp. 

*General*. Body small, stout, attenuated anteriorly. Mouth triangular with three lips, dorsal lip bearing two prominent cephalic papillae, two ventro-lateral lips bearing each one cephalic papillae and an amphid, with eight body papillae surrounding the cephalus. Three lips opening into oesophagus. Oesophagus divided into three parts: pharynx, cylindrical corpus and oesophageal bulb. Lateral alae beginning anterior to nerve ring and terminating anterior to cloaca. Nerve ring encircling oesophagus at mid-length. Excretory pore anterior to oesophageal bulb. Tail rounded with straight elongated end in females and curved ventrally in males, evenly narrowing with short process on tip.

*Male*. Measurements based on holotype and three paratypes. Body (Fig. 2a) 1.77–2.13 (1.97) [2.03] mm long, 177–203 (190) [178] wide at mid-body level. Lateral alae beginning at 122–216 (182) [122] from apex. Oesophageal pharynx 12–20 (15) [14] long and 16–22 (19) [16] wide; corpus 267–326 (299) [314] long and 27–42 (34) [27] wide; and oesophageal bulb 61–76 (67) [61] long and 70–84 (78) [70] wide. Nerve ring at 91–239 (155) [195] and excretory pore at 328–369 (354) [357] from apex. Tail bluntly rounded, 130–162 (149) [150] long, bearing short process at tip 9–15 (12) [15] long. Gubernaculum (Fig. 2e) Y-shaped with well sclerotised edges, 100–108 (102) [103] long. Spicules equal in shape, evenly narrowing with sharpened tips, observed without dissection of tail. Left spicule 87–96 (92) [87] long, right one 92–107 (97) [93] long. Fifteen [16] post-cloacal papillae (Fig. 2b) located in the tail region. Five pairs of plectanes (Fig. 2f, g) located anterior to cloaca, each bearing five to six tubercles directed posteriorly. In total about 146 papillae observed on entire body.

*Female*. Measurements based on allotype and 13 paratypes. Body (Fig. 2h) 2.24–4.24 (3.31) [3.18] mm long, 154–332 (250) [273] wide at mid-body level. Lateral alae (Fig. 2d) 138–293 (212) [138] from apex. Oesophageal pharynx 25–35 (30) [29] long and 38– 67 (57) [57] wide; cylindrical corpus 368–469 (410) [371] long and 35–68 (51) [48] wide and oesophageal bulb 81–124 (103) [98] long and 86–146 (125) [119] wide. Nerve ring at 110–184 (155) [130] and excretory pore at 352–477 (410) [352] from apex. Vulva at 1.22–2.07 [1.67] mm from anterior end of body (about 55% of body length), small, transversely slit with poorly developed lips. Tail 308–455 (371) [375] long, bluntly rounded, with long point.

### Taxonomic summary

Family Cosmocercidae Travassos, 1925.

Subfamily Cosmocercinae Railliet, 1916.

Genus *Cosmocerca* Diesing, 1861.

Species: *Cosmocerca monicae* n. sp.

Type-host: Senegal running frog *Kassina senegalensis* Dumeril et Bibron, 1841 (Amphibia: Anura: Hyperoliidae).

Type-locality: Daly farm, Potchefstroom, North-West Province, South Africa; coordinates: − 26.650324/27.062422.

Other localities: Louis Trichardt, Limpopo Province, South Africa; Makhado, Limpopo Province, South Africa.

Type-material: Holotype (male, [648]), allotype (female, [649]), paratypes (three males, 14 females, [650–664]) deposited in the National Museum Parasite Collection (Bloemfontein, South Africa).

Site in host: Large intestine.

Infection parameters: intensity: 4 – 33 (13.9); prevalence: 100% (all 19 studied frogs were infected); abundance: 13.9.

Etymology: The species is named after Monica Harnoster for her support throughout the investigation.

*Remarks.*

The new species belongs to the genus *Cosmocerca* due to the possession of plectanes, numerous papillae along the body and sexual dimorphism, since males are half the size of females (Baker 1980).

*Cosmocerca monicae* n. sp. differs from *C. ornata* in possessing 13 pairs of post-cloacal papillae, whereas the maximum number reported on *C. ornata* was 14 pairs. *Cosmocerca monicae* n. sp. differed from *C.* *daly* n. sp. in the shape of the gubernaculum: *C. monicae* n. sp. (Fig. 4D–F) has a Y-shaped gubernaculum, while *C.* *daly* n. sp. (Fig. 4A–C) has a V-shaped gubernaculum with hook-like structures on the margins. *C. monicae* n. sp. has 13 pairs of post-cloacal papillae, whereas *C.* *daly* has 14 pairs. Adult females of *C. monicae* n. sp. have an excretory pore located anterior to the level of the oesophageal bulb, whereas it is found posterior to the level of the oesophageal bulb in *C.* *daly* n. sp. *Cosmocerca monicae* n. sp. (Fig. 4D–F) differs from *C. makhadoensis* n. sp. in possessing a V-shaped gubernaculum, whereas *C. makhadoensis* n. sp. (Fig. 4G–I) has a club-shaped gubernaculum. The plectanes of *C. monicae* n. sp. are clearly visible on the lateral and ventral views, whilst the plectanes of *C. makhadoensis* n. sp. have almost transparent edges and are inconspicuous on the ventral view.

#### *Cosmocerca makhadoensis* n. sp.

*General*. Body small, stout, attenuated anteriorly. Mouth triangular with three lips, dorsal lip bearing two prominent cephalic papillae, two ventro-lateral lips each bearing two cephalic papillae. Three lips opening into oesophagus. Oesophagus divided into three parts: pharynx, cylindrical corpus and oesophageal bulb. Lateral alae beginning anterior to nerve ring and terminating anterior to cloaca. Nerve ring at mid-length of the of corpus. Excretory pore anterior to oesophageal bulb. Tail straight in females and curved ventrally in males, evenly narrowing with short process on tip.

Male. Body (Fig. 3a) 1.50–2.29 (1.72) [1.53] mm long, 122–172 (142) [149] wide at mid-body level. Lateral alae at 81–160 (115) [110] from apex. Oesophagus divided into three parts: pharynx 16–22 (20) [16] long and 15–21 (18) [17] wide; corpus 317–368 (339) [317] long and 24–31 (28) [31] wide; oesophageal bulb 67–76 (71) [61] long and 70–88 (78) [70] wide. Nerve ring at 101–163 (124) [104] and excretory pore at 268–344 (313) [299] from apex. Tail bluntly rounded 134–166 (152) [134] long, bearing short process at tip 13–23 (19) [17] long. Gubernaculum (Fig. 3e) club-shaped, having a narrowed small bulb at anterior part and well sclerotised edges, 98–110 (105) [105] long. Spicules equal in shape, evenly narrowing with sharpened tips, observed without dissection of tail. Left spicule 38–95 (71) [95] long, right one 41–103 (75) [103] long. Fourteen post-cloacal papillae (Fig. 3) in tail region. Five pairs of plectanes (Fig. 3f, g) almost transparent when observed on ventral view with indistinguishable margins, well visible on lateral view, each bearing from five to six trabeculae. In total about 108 papillae observed on entire body.

Female. Body (Fig. 3h) 2.8–3.9 (3.2) [3.0] mm long, 230–420 (302) [293] wide at mid-body level. Lateral alae (Fig. 3c) at 116–367 (291) [272] from apex. Oesophagus divided into three parts: pharynx 23–47 (33) [27] long and 32–58 (44) [39] wide; cylindrical corpus 355–776 (417) [361] long and 36–59 (49) [48] wide and oesophageal bulb 97–129 (113) [110] long and 118–165 (133) [128] wide. Nerve ring 118–244 (174) [195] and excretory pore 362–454 (403) [362] from apex. Vulva small slit, lips poorly developed at mid body level 1.3–1.9 (1.6) [1.5] mm from apex. Tail 131–486 (408) [389] long with short point.

### Taxonomic summary

Family Cosmocercidae Travassos, 1925.

Subfamily Cosmocercinae Railliet, 1916.

Genus *Cosmocerca* Diesing, 1861.

Species: *C*. *makhadoensis* n. sp.

Type-host: *Phrynomantis bifasciatus* Smith, 1847 (Amphibia: Anura: Microhylidae).

Type-locality: Makhado, Limpopo Province, South Africa; coordinates: 23.11010 / 29.90837.

Type-material: Holotype (male, [620]), allotype (female, [621]), paratypes (three males, 29 females, [622–647]) deposited in the National Museum Parasite Collection (Bloemfontein, South Africa).

Site in host: Large intestine.

Infection parameters: intensity: 4–136 (29.9); prevalence: 89.5% (17 of 19 were infected); abundance: 26.7.

Etymology: The species is named after the type locality.

*Remarks.*

The new species belongs to the genus *Cosmocerca* due to the possession of plectanes, numerous papillae along the body and sexual dimorphism, since males are half the size of females (Baker 1980).

*Cosmocerca makhadoensis* n. sp. differs from *C. ornata* in the shape of the gubernaculum, which is club-shaped in *C. makhadoensis* n. sp., while the gubernaculum of *C. ornata* is Y-shaped. Moreover, the plectanes of *C. makhadoensis* n. sp. are almost transparent when observed from the lateral parts and almost inconspicuous from the ventral view, in contrast to the prominent plectanes of *C. ornata*.

*Cosmocerca makhadoensis* n. sp. differs from *C.* *daly* n. sp. in the shape of the gubernaculum: *C. makhadoensis* n. sp. (Fig. 4g–i) has a club-shaped gubernaculum, while *C.* *daly* n. sp. (Fig. 4a–c) has a V-shaped gubernaculum with hook-like structures on the margins. Additionally, *C.* *makhadoensis* n. sp. has somatic papillae spread evenly over the entire body, while the papillae of *C.* *daly* n. sp. are located only on the lateral parts of the body. The plectanes of *C. makhadoensis* n. sp. are found to be almost transparent on the lateral view and almost inconspicuous in the ventral view, while those of *C.* *daly* n. sp. were clearly visible. Mature females of *C. makhadoensis* n. sp. have an excretory pore anterior to the level of the oesophageal bulb, whereas it is found posterior to the level of the oesophageal bulb in *C.* *daly* n. sp.

*Cosmocerca makhadoensis* n. sp. differs from *C.* *monicae* n. sp. in possession of a club-shaped gubernaculum (Fig. 4g–i), whereas *C. monicae* n. sp. (Fig. 4d–f) has a V-shaped gubernaculum. The plectanes of *C. makhadoensis* n. sp. are found to be almost transparent in the lateral view and almost inconspicuous in the ventral view, while the plectanes of *C. monicae* n. sp. are prominent and clearly visible.

### Molecular analyses

Sequences of the ITS-28S region of *C.* *daly* n. sp., *C. monicae* n. sp. and *C. makhadoensis* n. sp. were generated in this study. The fragments were trimmed to the shortest sequence, that of *C.* *daly* n. sp., which comprised 950 bp. Based on pairwise analysis of the ITS-28S fragments, the genetic distance between *C. daly* n. sp. and *C. monicae* n. sp. comprised 27 bp, 2.9%; between *C.* *daly* n. sp. and *C. makhadoensis* n. sp. 27 bp, 2.9% and between *C. monicae* n. sp. and *C. makhadoensis* n. sp. 10 bp, 1%.

**Fig. 5:**
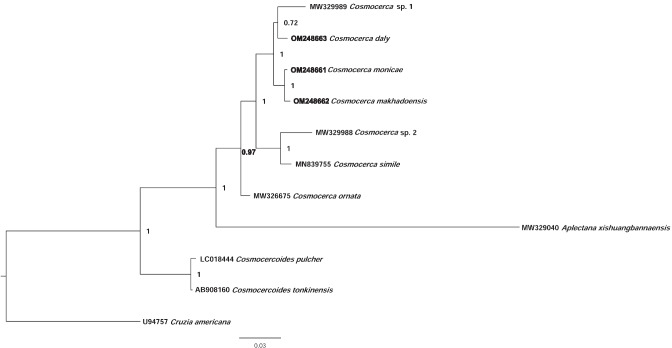
Phylogenetic tree based on Bayesian analysis of 739-bp- long alignment of 10 species of cosmocercid nematodes

Phylogenetic analysis based on the partial ITS-28S sequences (Fig. 5) showed distinctness of the clade formed by seven species of *Cosmocerca*, in which *C. ornata* appeared basal to the subclade formed by six other species. The three new species appeared in the separate branch in the subclade with *C. monicae* n. sp. and *C. makhadoensis* n. sp. grouping together and *C. daly* n. sp. grouping with *Cosmocerca* sp. 1 (parasite of *Hoplobatrachus rugulosus* (Wiegmann, 1834) in China). *Aplectana xishuangbannaensis* Chen, Gu, Ni et Li, 2021, as well as two species of *Cosmocercoides* Wilkie, 1930, formed separate lineages basal to the clade of *Cosmocerca* spp.

## Discussion

Currently 30 species of *Cosmocerca* are considered valid. This list is compiled by Sou et al. (2018), and also by Bursey et al. (2015), where it was organised by biogeographical region. Although there are no records for species of *Cosmocerca* from the Afrotropical region in the list compiled by Bursey et al. (2015), *C. ornata* has previously been recorded in South Africa (Baker 1981; Halajian et al. 2013). Unfortunately, no images, detailed descriptions or molecular data were provided. In our opinion, the previous records of *C. ornata* from South Africa might be the result of misidentifications and the found specimens might belong to one of the three species described herein or another new species.

Morphological differentiation of *Cosmocerca* is mostly based on the differences in male genitalia. All species described in the present study differed from *C. ornata* and from each other based mostly on the shape of the gubernaculum and somatic papillae. Females of *C.* *daly* n. sp. can be distinguished from the two other species by the position of the excretory pore, which is posterior to the level of oesophageal bulb, although immature specimens of *C.* *daly* n. sp. have an excretory pore at about the same position as two other species. Since females of most *Cosmocerca* spp. are rather indistinguishable and males are very scarce in samples (13 to 74 of the whole sample in all three species), molecular approaches are necessary for future identifications.

The molecular data of ITS-28S region confirmed the difference between the species. The difference in the ITS-28S between *C.* *daly* n. sp. and *C. makhadoensis* from the same locality was found to be 2.9% and 1% between *C. monicae* n. sp. and *C. makhadoensis* n. sp. from two distant localities. Of the available sequences in GenBank, we could use only one species of *Aplectana*, two of *Cosmocercoides* and four of *Cosmocerca* that overlapped with sequences obtained in the present study. In accordance with two recently published phylogenies of cosmocercids (Chen et al. 2020, 2021), our analysis showed closer relationships between *Cosmocerca* and *Aplectana* and their more distant relationship with *Cosmocercoides*. In our analysis, only a short fragment of a single nuclear marker of a few species was used and thus our analysis can be considered preliminary until more markers of more species around the globe are used.

All three species were found only in their host types and regardless of the presence of *C. boetgeri* and *K. senegalensis* at the same spot only their specific nematode species were collected. Similarly, only *C. makhadoensis* n. sp. was found in *P. bifasciatus* and no other cosmocercids were recorded from this host. Therefore, we suppose that the host specificity of *Cosmocerca* might be higher than it has been previously considered, and the presence of *C. ornata* throughout different frogs in South Africa is rather doubtful. We believe that this fact will be confirmed following the study of *Cosmocerca* from different hosts using morphological and molecular approaches.
